# Self-Sterility in *Camellia oleifera* May Be Due to the Prezygotic Late-Acting Self-Incompatibility

**DOI:** 10.1371/journal.pone.0099639

**Published:** 2014-06-13

**Authors:** Ting Liao, De-Yi Yuan, Feng Zou, Chao Gao, Ya Yang, Lin Zhang, Xiao-Feng Tan

**Affiliations:** Key Laboratory of Cultivation and Protection for Non-Wood Forest Trees, Ministry of Education, The Key Lab of Non-Wood Forest Products of Forestry Ministry, Central South University of Forestry and Technology, Changsha, Hunan, China; United States Department of Agriculture, United States of America

## Abstract

In this report, self-sterility in *Camellia oleifera* was explored by comparing structural and statistical characteristics following self-pollination (SP) and cross-pollination (CP). Although slightly delayed pollen germination and pollen tube growth in selfed ovaries compared to crossed ovaries was observed, there was no significant difference in the percentages of pollen that germinated and pollen tubes that grew to the base of the style. There was also no difference in morphological structure after the two pollination treatments. However, the proportions of ovule penetration and double fertilization in selfed ovules were significantly lower than in crossed ovules, indicating that a prezygotic late-acting self-incompatible mechanism may exist in *C. oleifera*. Callose deposition was observed in selfed abortive ovules, but not in normal. Ovules did not show differences in anatomic structure during embryonic development, whereas significant differences were observed in the final fruit and seed set. In addition, aborted ovules in selfed ovaries occurred within 35 days after SP and prior to zygote division. However, this process did not occur continuously throughout the life cycle, and no zygotes were observed in the selfed abortive ovules. These results indicated that the self-sterility in *C. oleifera* may be caused by prezygotic late-acting self-incompatibility (LSI).

## Introduction

*Camellia oleifera* (Theaceae), an evergreen shrub species, is widely cultivated in southern China. Oil from its seeds is commonly used as an edible oil with known health benefits. At this time, there are approximately three million hectares of cultivated area of *C. oleifera* in China, with 35.2 kg of oil per acre [1]. The large number of flowers with relatively low fruit/seed sets is a serious problem and has restricted development of the oil tea industry [1]. To address this issue, previous studies focusing on flowering biology [2], pollination biology [2], pollen viability [3]–[6], flower bud differentiation [7], megaspore and microspore development [8], male and female gamete development [8], and embryological development [9] have been performed in *C. oleifera*. However, these studies did not explore flower development or the large number of ovules that fail to mature into seeds during fruit development in nature [1], [9].

Self-sterility is a common reproductive phenomenon in Theaceae, which is mainly the result of self-incompatibility (SI) and early-acting inbreeding depression (EID) [10]–[16]. SI, an initiative abortion process, can prevent reproduction after selfing and enhance heterozygosity in plants [15]–[17]. SI is involved in several processes, including pollen inhibition on the stigma or in the style in some species of Brassicaceae, Polemoniaceae, Rosaceae, and Solanaceae [17]–[21]. Another type of SI that occurs in the ovary is called late-acting SI (LSI) or ovarian SI [17], [22]. Abortion in LSI may occur before or after zygote formation, and postzygotic mechanisms are more complicated than prezygotic mechanisms. It is known that prezygotic SI occurs in *Theobroma cacao* [23], *Acacia retinodes* [24], and *Camellia sinensis* [25], while postzygotic SI occurs in *Caesalpinia calycina* [26], *Pseudowintera axillaris* [27], *Tabebuia* [28], *Citrus grandis* Osbeck [29], and *Xanthoceras sorbifolium* [30]. In contrast, EID is caused by the expression of recessive alleles during seed development [17], [22]. This may occur throughout the plant life cycle, and aborting ovules/seeds may be observed in ovaries/fruits. It is currently difficult to distinguish between postzygotic SI and EID mechanisms in the study species.

In this report, we compared pollen germination, pollen tube growth, ovule penetration, double fertilization, embryonic development, and fruit/seed sets with a focus on morphology and anatomical structures following SP and CP. In addition, we characterized a possible mechanism of self-sterility in *C. oleifera*.

## Materials and Methods

### Ethics Statement

No specific permits were required for the described field studies, and the field observations did not collect any animal, endangered or protected specimen.

### Plant material

Populations of two *C. oleifera* cultivars (‘Xianglin XLC15′ and ‘Xianglin XLJ14′) were used in this study. The plants were mixed planted in Taoshui (27°05′06"N, 113°13′11"E), You County, Zhuzhou, Hunan Province, China. ‘Xianglin XLC15′ bloomed from late October to mid-December, with about 70 mm in diameter of the flower, while *C. oleifera* ‘Xianglin XLJ14′ bloomed from late October to late November, and the diameter of the flower was about 55 mm. Both of them were hermaphroditic and homogamous. Fruits of both cultivars matured in mid-to-late October of the next year [31]. The plants were 2.2-m high and 12-years-old, they were situated at 150 m above sea level on red soil and subjected to a typical subtropical moist climate. The mean annual temperature was 17.8°C, and the mean annual rainfall was about 1410 mm. Rainfall occurred primarily from April to June, accounting for 45% of the yearly total.

### Pollination treatments

Controlled pollination included an SP combination (‘Xianglin XLC15′× ‘Xianglin XLC15) with pollen from a mix of several individuals from the same clone and a CP combination (‘Xianglin XLC15′× ‘Xianglin XLJ14′) that was performed in November of 2011. Moreover, non-pollination (NP) was included. In addition to the three pollination treatments (SP, CP and NP), a control open-pollinated (OP) sample was also included to evaluate fruit and seed sets 1 year after pollination (AP). In SP and CP, the flowers were emasculated and pollinated during the bud stage so that the stigmas were not stained by any pollen until they became receptive. In NP, the stamens of the flower buds were picked before anthers shed pollens. Afterwards, all the pistils were bagged in sulfate paper bags for 7 days after pollination (DAP) and marked with signs. In OP, no action except signing the bud randomly was being taken. Self- and cross-pollinated pistils were harvested at 2, 4, 8, 12, 24, 36, 48, 60, 72, 84, 96, 108, and 120 h, and further every day from 6–10 DAP, every 2 days from 10–30 DAP, every 5 days from 30–50 DAP, and every 20 days from 50–210 DAP, with 15 pistils collected at each time point. Unpollinated and bagged samples were harvested every 2 days from the beginning of the study until the 30th day, then every 20 days until they dropped off, with 10 pistils harvested each time. All collected samples were fixed in formalin: acetic acid: ethyl alcohol (5: 5: 90, V/V) and stored at 4°C prior to sectioning [32]. In each pollination combination, 1000 flowers were pollinated and used for fluorescence analysis and sectioning in the SP and CP experiments. 300 non-pollinated flowers were used for sectioning and statistics. In addition to the previously explained pollination, other 300 pollinated flowers with three replicates (each 100 flowers) were used to investigate fruit and seed sets after SP, CP, and NP.

### Pollen germination, pollen tube growth, and ovule penetration following CP and SP

Pollen germination was observed using the method of Hiratsuka et al. [33] with minor modifications. Briefly, the style 2–120 h after SP and CP was macerated in 8 M NaOH for 3–4 h to soften the materials, after which it was thoroughly washed with deionized water several times with no residual chemical substances. The samples were stained for at least 4 h in 0.05% aniline blue dissolved in 0.15 M K_2_HPO_4_. The stigma was placed on a glass slide and then slowly flattened with a cover slip, as described by Chen et al. [25]. The length of the pollen tubes was measured using Motic Images Plus 2.0 software. Pollen germination and pollen tube growth were observed and photographs were taken using a fluorescence microscope (Olympus BX-51, Tokyo, Japan).

Ovule penetration into the embryo sac in both the CP and SP experiments was examined from 60 h to 25 DAP through sectioning and aniline blue staining. 3–5 ovaries which contained 16–25 ovules each ovary were observed per sample. Aniline blue staining was performed as described above. Each slice was cut to 15 µm. Several serial sections in one ovule were stained with aniline blue for 1 h after eliminating the paraffin adhered to the glass slide to detect callose [34]. Ovule penetration into the embryo sac was demonstrated based on the presence of pollen tubes penetrating the micropyle [17]. An Olympus BX-51 fluorescence microscope was also used to observe and take photos of penetrating ovules.

### Double fertilization, embryo sac, and ovule development following CP and SP

Ovaries between 60 h and 210 days after SP and CP, and ovaries at all stages following NP were dehydrated in ethyl alcohol, embedded in paraffin with a 58–60°C melting point, sectioned every 10 µm, and stained with hematoxylin-eosin Y to detect double fertilization, embryo sacs, ovules, and seed development [35]. Double fertilization was identified based on the presence of a degenerated and intensively colored synergid or the formation of endosperm nuclei, as well as the presence of zygotes or embryos [36]. To assess the area, as well as the thickness of the inner and outer integuments, we examined stained sections in the center of the ovary using Motic Images Plus 2.0 software, by which quantitative differences between various stages following CP, SP, and NP could be determined. Microscopic observations and photography were performed as described above.

### Fruit set, seed set, and fruit characteristics

SP, CP, OP, and NP were performed to evaluate the fruit set, seed set, and fruit characteristics. The number of subsistent fruits and seeds were counted. Seeds were considered fertile if they contained a complete seed composition and were full in appearance compared to incomplete, flat, and sterile seeds. The seed set was then calculated as the number of fertile seeds divided by the number of total ovules in mature fruits. Pollinated flowers that failed to form mature fruits or fell off before maturity were not included in the seed set (but were included in the fruit set) [37]. The fruit characteristics examined included mean single fruit transverse diameter, vertical diameter, and weight. The index of self-incompatibility (ISI) was used to assess the degree of SI in *C. oleifera*, which was calculated by dividing the seed set after SP by the set after CP [38]–[40].

### Statistical analysis

The SPSS statistical package, version 19.0, was used for most of the statistical analysis. Comparative variables between the CP and SP treatments, including pollen tube length, percent of pollen germination on the stigma, no. of pollen tubes in the style, percent of pollen tubes at the style base, percent of penetrated ovules, and ovule size, were examined. Comparisons were performed using a one-way ANOVA with 95% confidence interval to determine whether there were any significant differences in pollen tube length, percentages of pollen and pollen tubes at various stages, double fertilization and ovule development after SP and CP. Comparisons of the mean area of embryo sacs, fruit set, seed set and fruit characteristics after four pollination treatments were evaluated based on Duncan's multiple range test at the 5% level. Statistical data with 0 were read as N/A and they were treated as missing values when statistical analysis. All linear and scatter plots were generated using Origin 8.5 software. The Design Expert software, version 8.0, was used to evaluate the effects of three fertilization treatments, the time after pollination, and their interaction in integument thickness with the general factorial method. The factor of DAP was treated as a quantitative factor, and the fertilization treatment as a qualitative factor, thus, a 1-factor response surface analysis was generated.

## Results

### Characterization of pollen germination, pollen tube growth, and ovule penetration following SP and CP

**Figure 1:**
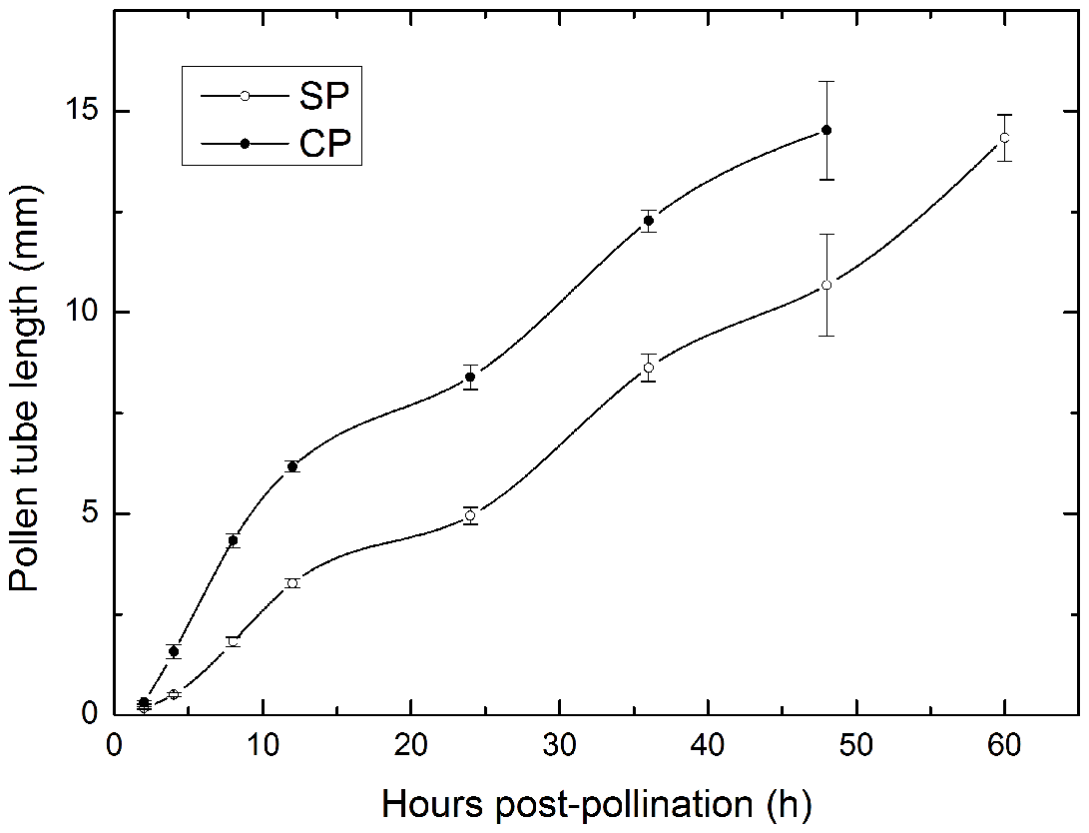
Mean length of pollen tubes at various times following SP and CP. Vertical bars represent the standard deviation.

**Figure 2:**
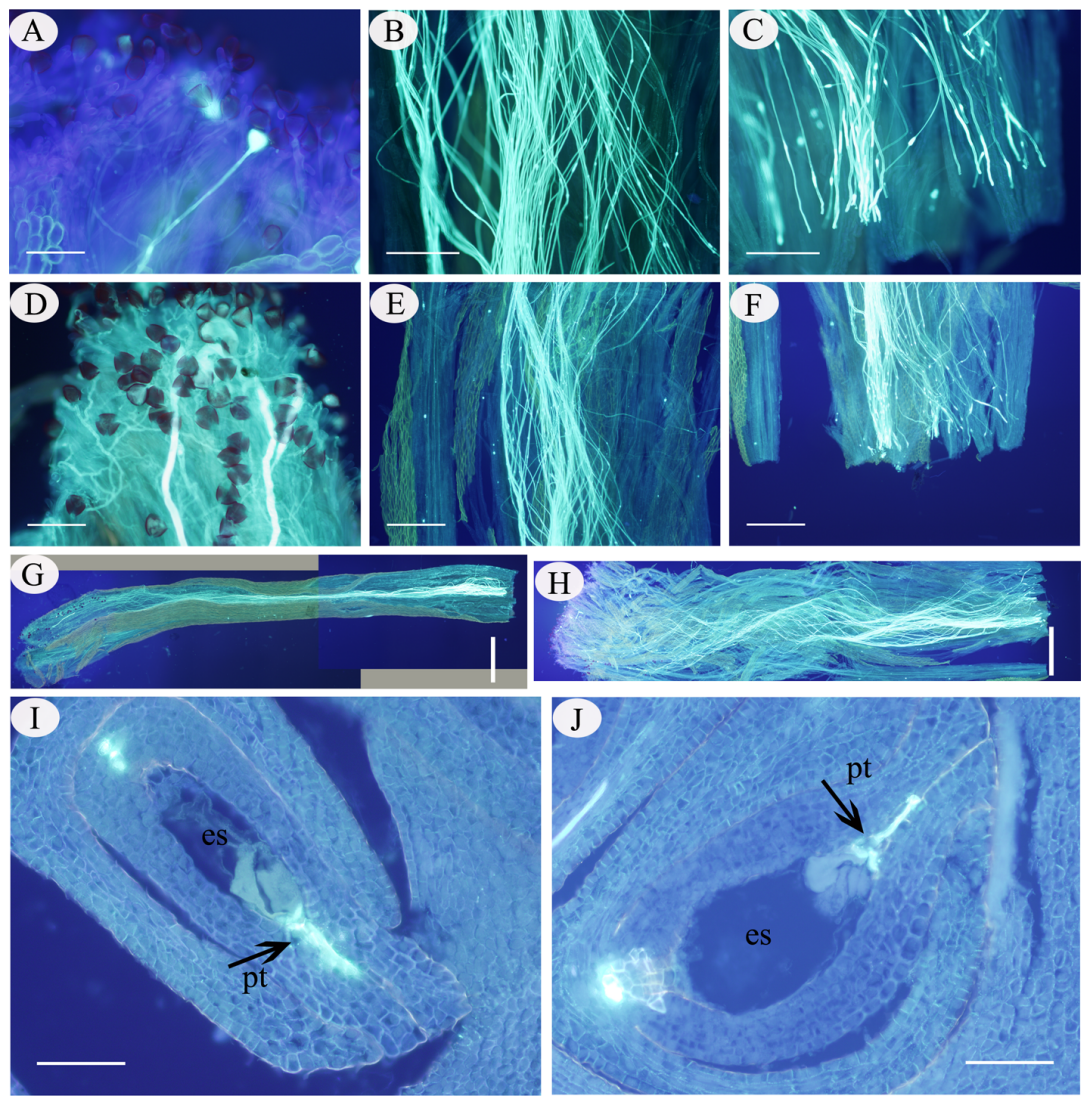
Pollen germination, pollen tube growth, and ovule penetration after SP and CP in *C. oleifera*. (A). Pollen germination 2 h after SP. (B). Pollen tubes in the middle of the style 36 h after SP. (C). Pollen tubes at the base of style 60 h after SP. (D). Pollen germination 2 h after CP. (E). Pollen tubes in the middle of the style 24 h after CP. (F). Pollen tubes at the base of style 48 h after CP. (G). Overall growth 60 h after SP. (H). Overall growth 48 h after CP. (I). A pollen tube penetrating an embryo sac 84 h after SP. (J). Ovule penetration 60 h after CP. Abbreviations: es, embryo sac; pt, pollen tube. Bars: A, D, I, J = 200 µm; B, C = 500 µm; E, F = 1000 µm; G, H = 2000 µm.

Significant differences in pollen tube length at every stage over 2–48 h following SP compared to CP were observed in *C. oleifera*, although the length increased continuously during the life cycle (Fig. 1; 2 h AP, SS_B_ = 0.032, df = 1, F = 21.275, P<0.05; 4 h AP, SS_B_ = 1.696, df = 1, F = 104.585, *p*<0.05; 8 h AP, SS_B_ = 9.45, df = 1, F = 432.501, *p*<0.05; 12 h AP, SS_B_ = 12.586, df = 1, F = 793.236; *p*<0.05; 24 h AP, SS_B_ = 17.785, df = 1, F = 257.13, *p*<0.05; 36 h AP, SS_B_ = 19.984, df = 1, F = 211.022, *p*<0.05; 48 h AP, SS_B_ = 22.157, df = 1, F = 14.31, *p*<0.05). However, no morphological or structural differences were observed in pollen germination and pollen tube growth in the style following SP and CP. At 2 h AP, a small number of selfed pollen grains had germinated compared with the large number of pollen grains that germinated 2 h after CP (Fig. 2A, D). At 36 h after SP, the pollen tube length reached about ½ of the style length, while 24 h was required to reach this length following CP (Fig. 2B, E). Afterwards, it took approximately 60 h for selfed tubes to grow at the base of the style, and only 48 h for crossed tubes (Fig. 2C, F). The first selfed pollen tube penetrated the embryo sac at 84 and 60 h after SP and CP, respectively (Fig. 2I, J). The pollen grains could germinate normally and the pollen tubes could grow normally at the base of the style, and even penetrate the ovule, regardless of whether they were produced by SP or CP.

**Figure 3:**
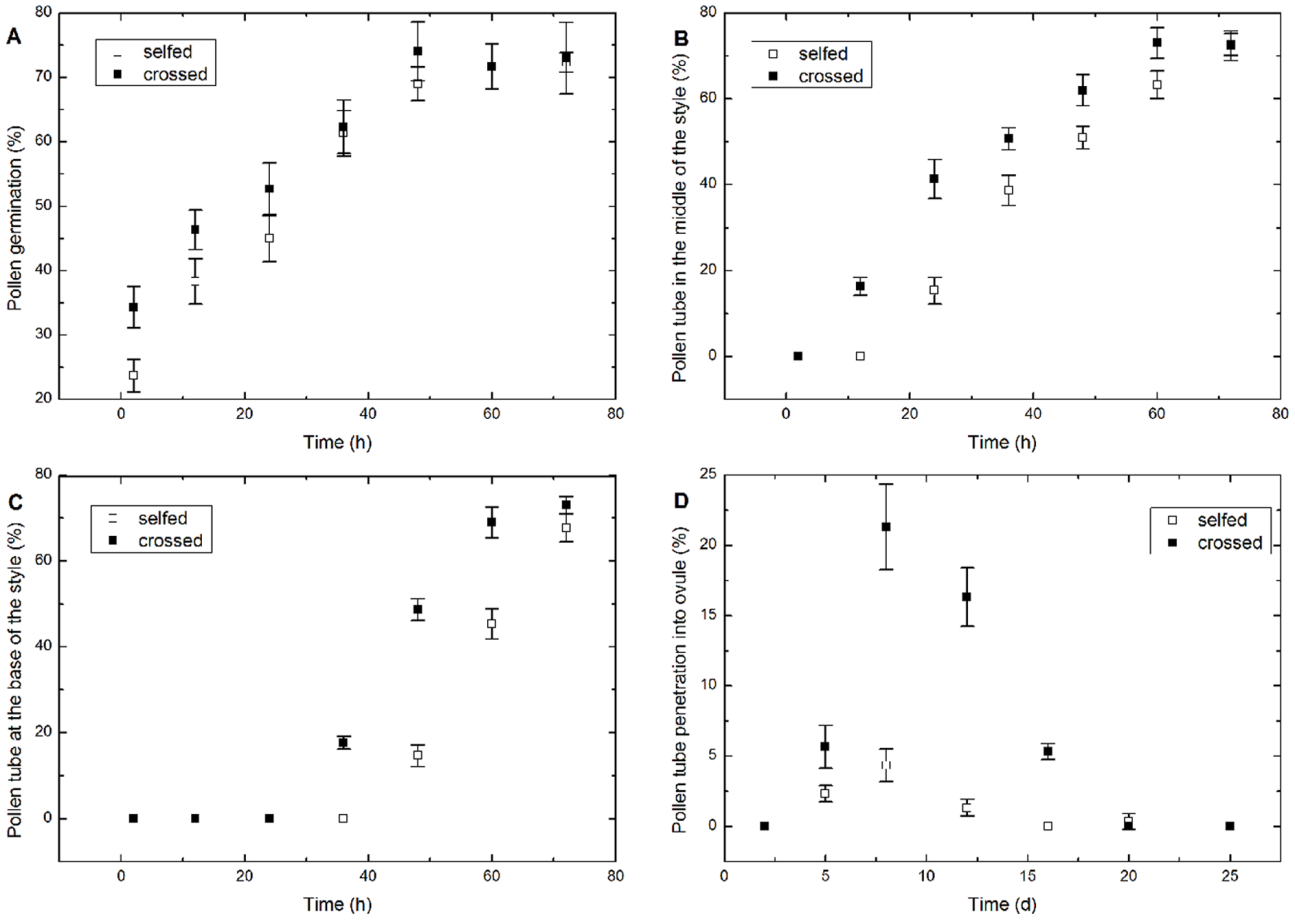
Percentages of pollen and pollen tubes at various stages after SP and CP. (A). Germinated pollen on the stigma. (B). Pollen tubes in the middle of the style. (C). Pollen tubes at the base of the style. (D). Pollen tubes penetrating into an ovule. Vertical bars represent the standard deviation.

Differences were observed based on a one-way ANOVA regarding the percentages of germinated pollen (Fig. 3A), pollen tubes in the middle of the style (Fig. 3B), pollen tubes at the base of the style (Fig. 3C), and penetrated ovules (Fig. 3D) at various times following SP compared to CP. With the increasing time AP, the percentages of germinated pollen, pollen tubes in the middle of the style, and pollen tubes at the base of the style increased initially, then they maintained a steady level. Nonetheless, the percentage of pollen tubes penetrating the ovule decreased significantly 8 DAP following both SP and CP (Fig. 3D). Pollen germination peaked at 72.3% 72 h after SP vs. 74% 48 h after CP. Significant differences in the percent of germinated pollen at 2 h (SS_B_ = 170.667, df = 1, F = 20.48, *p*<0.05) and 12 h (SS_B_ = 96, df = 1, F = 0.041, *p*<0.05) were observed following SP compared to CP, while no differences were observed at 24, 36, 48, 60, and 72 h (*p*>0.05 in these treatments). At 72 h AP (SS_B_ = 0.167, df = 1, F = 0.018, *p*>0.05), there were no significant differences in the percentage of pollen tubes in the middle of the style, similar to the percentage of pollen tubes at the base of the style (SS_B_ = 42.667, df = 1, F = 5.953, *p*>0.05, 72 h AP).

**Figure 4:**
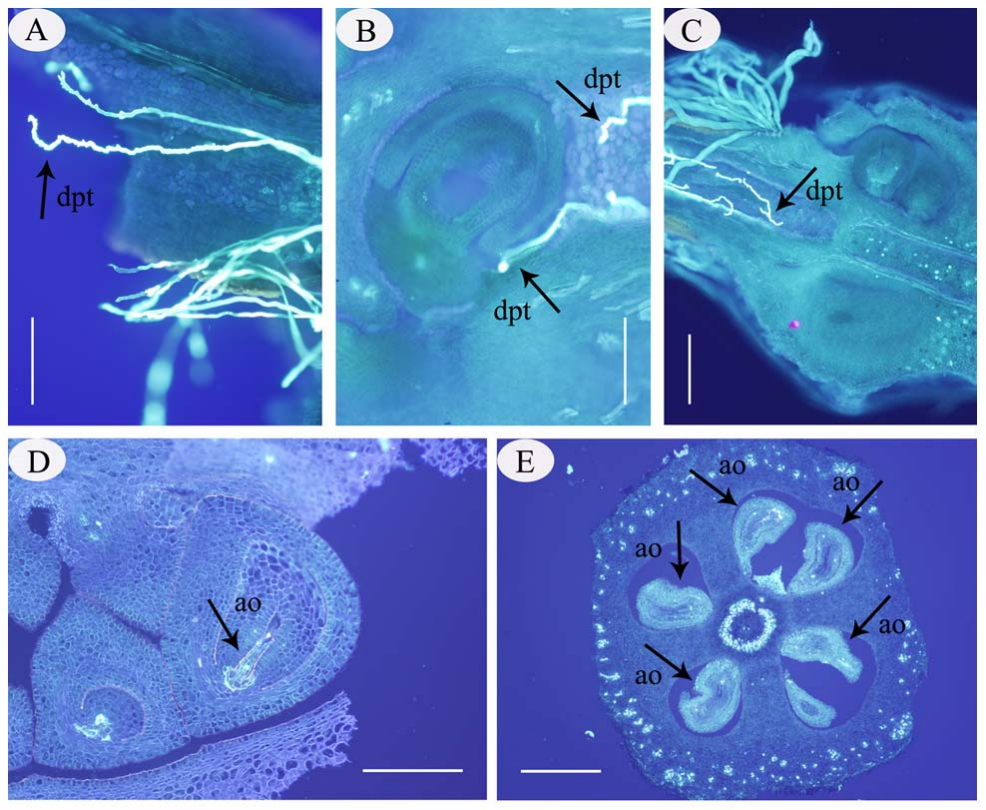
Distorted pollen tube grown in the ovary after SP in *C. oleifera*. (A). Distorted pollen tube with irregular ovaries 60 h after SP. (B). Distorted pollen tube with a reserving tube (the arrow above) and swelling tip (the arrow below) outside the micropyle 78 h after SP. (C). Distorted pollen tube with fortification of the ovaries 96 h after SP. (D). An abortive ovule with bright spots in the embryo sac 10 days after SP. (E). An abortive ovule with bright spots throughout the ovary 25 days after SP. Abbreviations: ao, abortive ovule; dpt, distorted pollen tube. Bars: A, B, D = 500 µm; C, E = 1000 µm.

Although the growth of pollen tubes in the style showed no structural differences between SP and CP, abnormal pollen tubes were observed in the ovary after SP (Fig. 4). In our study, distorted pollen tubes containing reversal tubes (Fig. 4B), swelling tube tips with callose deposits (Fig. 4B), irregular tubes (Fig. 4A), furcal tubes (Fig. 4C), and abortive ovules were observed in the SP samples. However, furcal tubes were the most common.

Nevertheless, the percentage of penetrated ovules was significantly greater following CP than SP. The pollen tubes entered embryo sacs through a degenerated synergid, and the other synergid disappeared after a period of time in normal ovules. Based on our observations, at 8 DAP the maximum ratio of penetrated ovules after SP was approximately 4.33±1.15% compared with 21.33±3.06% after CP (SS_B_ = 433.5, df = 1, F = 81.281, *p*<0.05).

### Double fertilization, embryo sac, and ovule development following SP and CP Double fertilization after SP and CP

**Figure 5:**
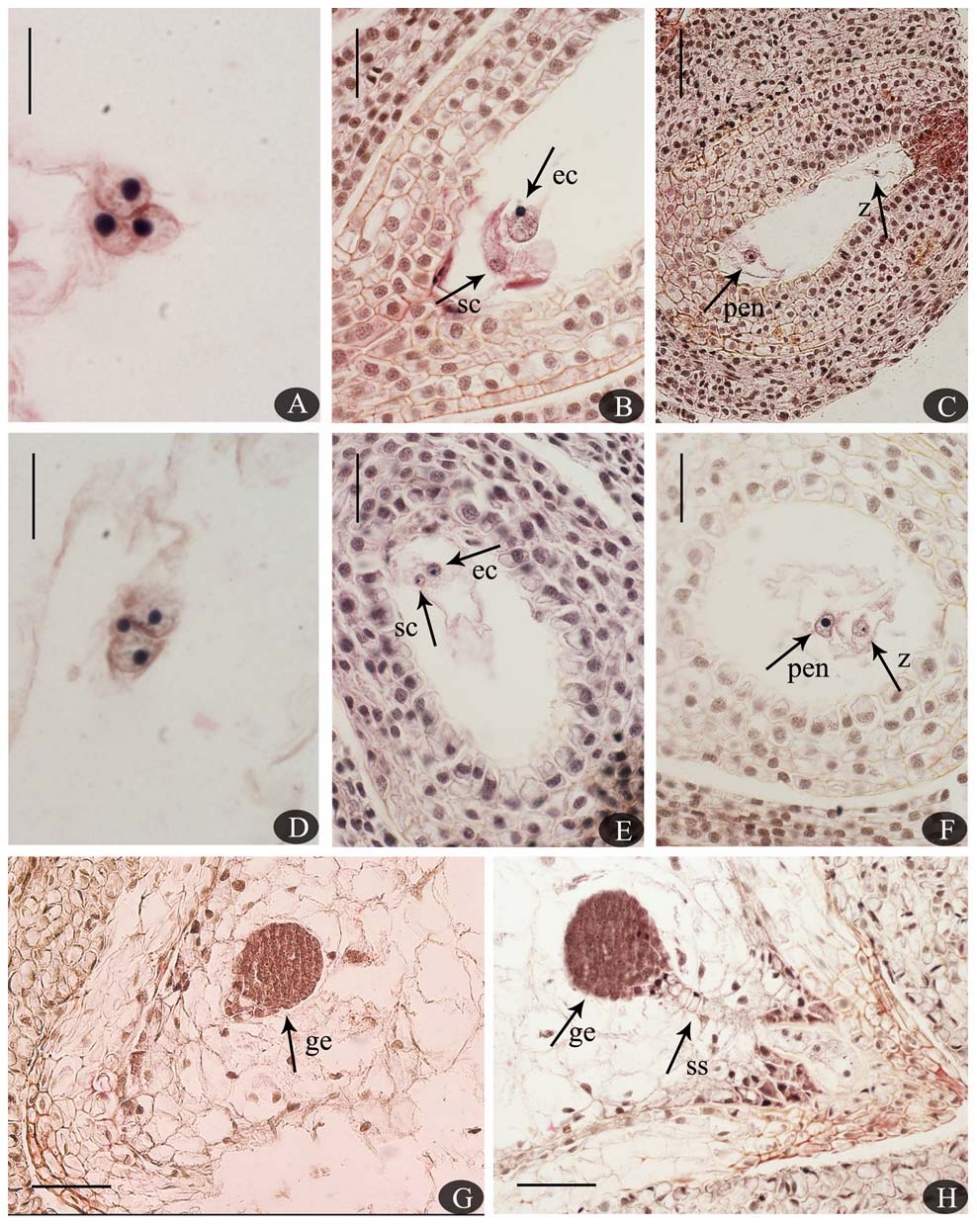
Normal double fertilization and early embryonic development after SP vs. CP in *C. oleifera*. (A). Polar nucleus fusion with a sperm nucleus 120 h after SP. (B). Sperm cell approaching an egg cell 96 h after SP. (C). A zygote and primary endosperm nucleus in an embryo sac 14 days after SP. (D). Polar nucleus fertilization 720 h after CP. (E). Sperm cell fused with an egg cell 6 days after CP. (F). A zygote and primary endosperm nucleus 10 days after CP. (G). A globular embryo 210 days after SP. (H). A globular embryo and suspensor 200 days after CP. Abbreviations: ec, egg cell; ge, globular embryo; pen, primary endosperm nucleus; sc, sperm cell; ss, suspensor; z, zygote. Bars: A, D = 1000 µm; B, E, F = 100 µm; C, G, H = 200 µm.

Double fertilization was demonstrated based on the fusion between sperm and egg cells or between a polar nucleus and sperm cells, as indicated by the presence of zygotes and a primary endosperm nucleus in embryo sacs after both SP and CP (Fig. 5). There were no structural differences between normal selfed and crossed embryos based on anatomical observations, but differences were observed in the frequency of double fertilization and ovule sterility (Fig. 5; Table 1). The highest ratio of double fertilization observed after SP (5.08%) was significantly lower than after CP (24.39%) at the same time AP, which corresponded to ovule penetration in SP compared to CP ovaries. After both SP and CP, delayed ovule penetration and double fertilization were observed in *C. oleifera* (although this was not statistically significant). SP resulted in a smaller proportion of double fertilization at all times compared with CP, suggesting that certain fertilization barriers existed. The level of sterility increased in 4 weeks AP. The process of double fertilization was rarely observed in selfed or crossed ovules, although a relatively higher percentage of double fertilization was observed after CP than SP.

**Table 1:** Anatomical and statistical details of ovule and seed development following self-pollination (SP) and cross-pollination (CP) at 1 week ovules, 2 weeks, 3 weeks, 4 weeks, and 1 year seeds after pollination.

			%				
Flower harvested	Pollination treatment	No. of samples observed	Normal ovules/seeds	Sterile ovules/seeds	Double fertilization	Zygote	Endosperm
1 week	SP	103	79.61	20.39	2.91	0.97	1.94
	CP	135	71.11	28.89	4.44	1.48	2.22
2 weeks	SP	142	65.49	34.51	3.52	0.70	1.41
	CP	104	66.35	33.65	24.04	11.54	7.69
3 weeks	SP	118	27.97	72.03	5.08	1.69	1.69
	CP	123	53.66	46.34	24.39	4.07	15.45
4 weeks	SP	181	29.83	70.17	4.42	1.10	2.21
	CP	113	46.90	53.10	18.58	5.31	10.62
1 year	SP	171	10.53	89.47	2.92	1.75	1.75
	CP	123	54.47	45.53	23.58	17.07	23.58

### Embryo sac area and integument thickness

To examine the irregular anatropous ovule in *C. oleifera*, the mean area and integument thickness (rather than ovule length and width) were measured. The mean embryo sac area (Table 2) and variance analysis of integument thickness (Table 3, Fig. S1) were examined at various stages following different pollination treatments. There were no significant differences in mean area between the selfed ovules and crossed ovules by 90 days, but significant differences were observed in the non-fertilized ovules, which indicates that ovule growth was determined based on whether fertilization occurred, rather than whether pollination occurred before zygote development. When the zygote began to divide, significant differences were gradually observed among self-, cross-, and non-fertilized ovules. Finally, the area of selfed embryo sacs was larger than the area of crossed embryo sacs. However, under the same pollination conditions, we observed an increase in both SP and CP selfed seeds and crossed seeds. Within the first 50 days, an increased embryo sac area in non-fertilized ovules was observed, while the area decreased 50 days after harvesting (with a final area of 0), indicating abortive ovules.

**Table 2:** The mean (±SD) area (µm^2^) of embryo sacs in self-fertilized (SF), cross-fertilized (CF), and non-fertilized (NF) ovules at various times.

DAP	SF	CF	NF
1	20789.91 (591.58) Ba	20608.85 (497.57) Ba	1944.27 (112.04) Aa
14	22849.99 (742.68) Bab	22204.87 (718.09) Bab	6111.42 (179.29) Ad
30	24529.11 (612.24) Bab	24069.40 (525.73) Bbc	14928.28 (301.08) Af
50	25829.85 (1352.91) Bab	26134.31 (1167.62) Bcd	17313.16 (211.37) Ag
70	25877.17 (518.59) Bab	26048.65 (292.39) Bcd	12807.90 (240.35) Ae
90	28603.46 (784.15) Bb	28295.16 (738.38) Bd	5250.70 (238.61) Ac
130	36884.32 (330.61) Cc	35226.40 (916.50) Be	3090.72 (235.74) Ab
170	111299.25 (8333.01) Bd	97441.08 (3406.03) Af	N/A

Different capital letters within the same line represent significant differences at the 5% level as determined by Duncan's multiple range test, while the same letter represents no significant differences. In addition, completely different small letters after capital letters within the same column represent significant differences at the 5% level based on Duncan's multiple range test, while the same small letter represents no significant difference at the 5% level. Abbreviation: DAP, days after pollination; SD, Standard Deviation.

**Table 3:** The ANOVA (IIT/OIT) for response surface of integument thickness (µm) in three fertilization treatments (FT) at various times.

Source	Sum of Squares	df	Mean Square	F Value	*p*-value Prob >F
Model	75077/25709	9/9	8342/2857	274/234	<0.0001/<0.0001
A-DAP	53514/24164	1/1	53514/24164	1760/1979	<0.0001/<0.0001
B-FT	2977/19	2/2	1488/9.47	49/0.78	<0.0001/0.465
AB	7535/50	2/2	3768/25	124/2.06	<0.0001/0.136
A^2^	3394/620	1/1	3394/620	112/51	<0.0001/<0.0001
A^2^B	3003/15	2/2	1502/7.25	49/0.59	<0.0001/0.555
A^3^	4654/841	1/1	4654/841	153/69	<0.0001/<0.0001
Residual	1885/757	62/62	30/12	N/A	N/A

*p*-value <0.05 represents significant differences at the 95% confidence interval based on response surface analysis. Abbreviations: DAP, days after pollination; IIT, inner integument thickness; OIT, outer integument thickness; FT, fertilization treatments.

Both DAP and FT had significant differences in inner integument thickness (*p*<0.0001) (Table 3). Additionally, significant differences were observed in the interaction of DAP and FT in the inner integument thickness (*p*<0.0001) (Fig. S2). The effect of DAP was significant in outer integument thickness (*p*<0.0001), similar to the inner integument. However, no significant differences in the outer integument thickness were observed in FT (*p*>0.05) and the interaction of DAP and FT (*p*>0.05) (Fig. S3). It meant that the presence or absence of fertilization, or the type of fertilization, did not affect the outer integument growth. Moreover, the F value of DAP was greater than FT both in the inner and outer integument thickness, which revealed that the DAP was a more important factor than FT in integument thickness.

### Ovule/seed abortion

**Figure 6:**
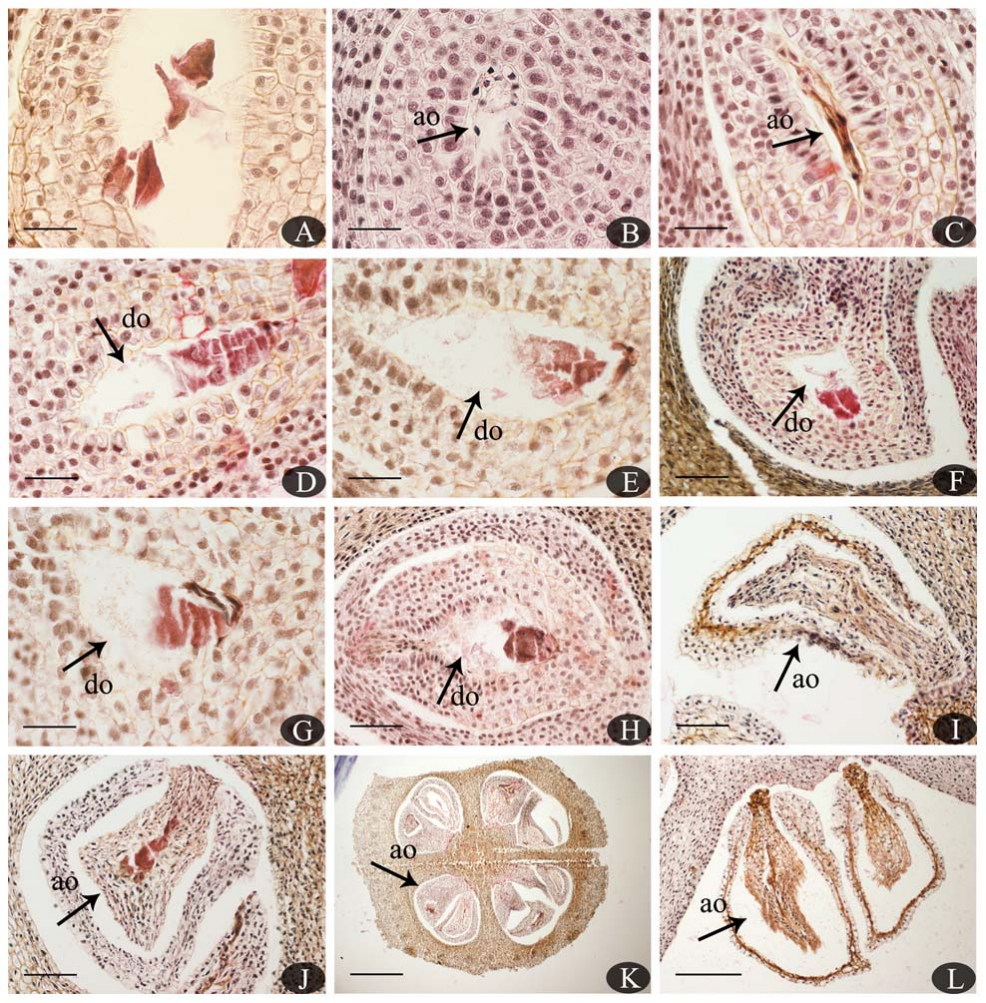
Degenerate and abortive ovules in self-pollinated ovaries at various stages. (A). Two sets of egg apparatus in embryo sacs 72 h after SP. (B–C). Abortive ovules with shriveling embryo sacs but normal development of the integument 8 and 10 days after SP, respectively. (D–H). Embryo sacs with an abnormal shape and degenerated egg apparatus 16, 20, 25, 30, and 35 days after SP, respectively. (I–L). Abortive ovules with dead whole tissues 40, 45, 50, and 170 days after SP, respectively. Abbreviations: ao, abortive ovule; do, degenerate ovule. Bars: A–E, G = 100 µm; F, H–J = 200 µm; K = 1000 µm; L = 500 µm.

A small number of SP vs. CP ovules developed to mature seeds normally through double fertilization. Ovules were mostly observed aborting during seed formation, especially prior to the zygote development phase (Fig. 6). Abortion was judged based on structural characteristics, with a strongly stained egg apparatus and abnormal embryo sacs. Ovules with shriveling embryo sacs but normal integument development (Fig. 6B, C) at early stages, embryo sacs with screwy shapes and a degenerated egg apparatus (Fig. 6D–H), shrinking ovules with dead whole tissues (Fig. 6I, J, L), and even completely dead ovaries (Fig. 6K) were observed at different stages following SP. Two sets of egg apparatus were observed in one of the selfed ovules 72 h after SP (Fig. 6A), but there was no evidence of polyembryony in mature seeds in this study, while Cao observed a small number of polyembryonic seeds in *C. oleifera* [9].

**Figure 7:**
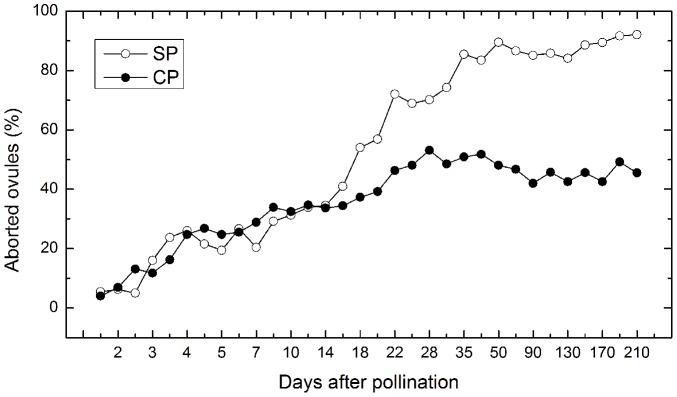
Percentage of aborted ovules 210 days after SP and CP at various stages.

Based on the paraffin section statistics of 214 ovaries and 4242 ovules after SP, and 180 ovaries and 3736 ovules after CP, the ratio of aborted ovules remained significant by 16 days after SP vs. CP, whereas it became distinct 16 days post-pollination (Fig. 7). The percentage of aborted ovules reached 85.5% 35 days after SP and 46.3% 22 days after CP, and was then relatively stable until the seeds reached maturity, which demonstrates that the greatest incidence of self-sterility occurred prior to zygote division. On the other hand, ultimately there was a higher proportion of seed abortion following SP than CP.

### Fruit set, seed set, and fruit characteristics following CP, SP, OP, and NP

**Figure 8:**
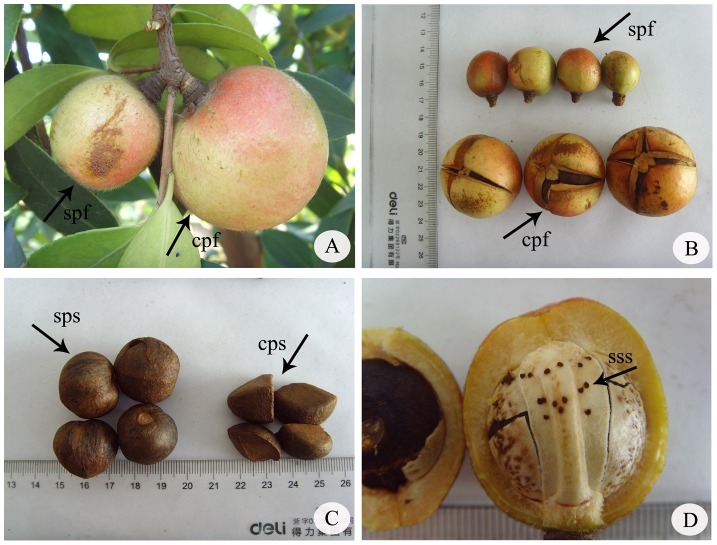
Mature fruits, mature seeds, and abortive seeds after SP and CP in C. oleifera. Abbreviations: cpf, CP fruit; cps, CP seed; spf, SP fruit; sps, SP seed; sss, self-sterile seed.

Comparisons between fruit sets, seed sets, and fruit characteristics after CP, SP, OP, and NP over time revealed quantitative differences (Table 4). Significant differences in mean fruit sets (SS_B_ = 2882.889, df = 2, MS_B_ = 1441.444, F = 123.552, *p*<0.05), seed sets (SS_B_ = 9448.32, df = 2, MS_B_ = 4724.16, F = 167.315, *p*<0.05), transverse diameter (SS_B_ = 2333.301, df = 2, MS_B_ = 1166.65, F = 43.366, *p*<0.05), vertical diameter (SS_B_ = 924.121, df = 2, MS_B_ = 462.06, F = 33.96, *p*<0.05), and weight (SS_B_ = 9660.556, df = 2, MS_B_ = 4830.278, F = 52.841, *p*<0.05) per fruit were observed following SP, CP and OP. Fruit size after CP was larger and heavier than after the other treatments, but seed size after SP was visibly larger than after CP (Fig. 8). Fruit size and weight for OP differed between SP and CP (Table 4). NP with no fruit revealed no apomixes in *C. oleifera*. Similarly, there were significant differences in the fruit sets among SP and CP. On the contrary, no significant differences were observed between SP and OP in fruit sets, indicating a high incidence of SP in nature. The ISI of *C. oleifera* was 0.19 (10.12/53.41). This corresponds to the most self-incompatible category according to Zapata and Arroyo's classification system [38], and may explain the high percentage of aborted ovules (corresponding to the high level of self-sterility) observed in the present study.

**Table 4:** Mean (±SD) comparisons of fruit set, seed set, and fruit characteristics of four pollination treatments.

Pollination treatment	Fruit set (%)	Seed set (%)	Transverse diameter (mm)	Vertical diameter (mm)	Weight (g)
SP	31.67 (2.52) a	10.12 (3.76) a	32.68 (7.70) a	30.72 (4.49) a	20.30 (12.54) a
CP	71.33 (3.51) b	53.41 (4.62) c	45.06 (2.33) c	38.55 (2.82) c	44.97 (7.02) c
OP	35.33 (4.04) a	35.22 (7.02) b	37.60 (3.99) b	34.16 (3.56) b	27.47 (8.22) b
NP	N/A	N/A	N/A	N/A	N/A

Different letters within the same column represent significant differences at the 5% level based on Duncan's multiple range test, while the same letter represents no significant differences. Abbreviations: SP, self-pollination; CP, cross-pollination; OP, open-pollination; NP, non-pollination; SD, Standard Deviation.

## Discussion

In this report, we demonstrated that *C. oleifera* ‘XianglinXLC15′ is a self-sterile cultivar since the fruit and seed sets after SP (pollen from the same genotype) were significantly reduced compared to after CP with a different cultivar. However, pollen tube growth to the ovary after selfing and the incidence of ovule penetration was indicative of LSI in selfed pistils. In addition, the significantly lower percentages of ovule penetration and double fertilization in selfed ovules vs. crossed ovules support a prezygotic LSI mechanism in *C. oleifera* ‘XianglinXLC15′. We then discussed the possible causes of self-sterility in detail in *C. oleifera* based on a comparison to other species with similar characteristics.

### Pollen tube inhibition and callose deposition in *C. oleifera*

Based on histological fluorescence microscopic observations of the pistil structure and a detailed analysis of the percentage of pollen tubes at different locations in pistils after SP compared to CP, no significant differences in morphological structure and the percentage of germinated pollen grains, pollen tubes in the middle of the style, or pollen tubes at the base of the style were observed. This was also reported for *C. sinensis*, with pollen tubes successfully reaching the ovary 48 h AP following both SP and CP [25]. In the present study, pollen tubes reached the base of the style 60 h after SP and 48 h after CP, and the growth speed of crossed tubes was slightly greater than of selfed tubes, similar to our previous findings in *C. oleifera* ‘Huashuo’ [6].

Furthermore, the majority of the pollen tubes did not penetrate into embryo sacs after SP. The present study shows that the percentage of penetrated ovules following SP was significantly lower than following CP. Also, a mass of bright spots considered to be callose was detected in the ovules after SP; this is probably related to ovule abortion, ovule non-fertilization, and ovule death [41]. The inhibition of pollen tubes has been reported in other species of the Theaceae. For example, Yang et al. reported inhibition at the base of the style [42]. Wang et al. also observed this phenomenon and suggested that parts of selfed tubes could grow to the ovary [43]. However, this was also documented in *Dolichandra cynanchoides* and *Tabebuia nodosa*, with pollen tubes growing successfully to the ovary with ovule penetration [44]. Based on our results, callose deposition was observed in abortive ovules 10 and 25 days etc. after SP. This was observed in a significant number of self-sterile species, with different degrees of fluorescence in abortive ovules but not in fertile ovules [45]. Li et al. reported that some *C. oleifera* cultivars were self-compatible since no callose deposition was observed [5]. In *C. sinensis*, callose deposition was not observed in ovules, but was observed outside the micropyle, indicating that the pollen tubes were prevented to penetrate micropyle [25].

### Mechanism of prezygotic SI in *C. oleifera*

Self-sterility in angiosperms results in a reduced fruit/seed set following SP compared to CP, and is mainly achieved through SI and EID [14], [15]. The abortion period was used to distinguish between SI and EID. For SI, uniform abortion occurred at a single stage during seed development, while continuously abortive development was indicative of EID [22], [27], [46]. Thus, if the self-sterility was caused by postzygotic SI, fertilized ovules would be consistently rejected, and uniform rejection and failure would be observed [22], [47], [48]. Self-sterility resulting from EID is expected to act at any stage during embryonic development [22], [37], [49].

In the present study, double fertilization in a small number of selfed ovules was demonstrated. The selfed ovules also showed a smaller proportion of double fertilization 3 weeks AP, revealing that some fertilized ovules had degenerated, similar to *Eucalyptus globules* [50]. For comparison, zygotes were not observed in whole abortive ovules. The presence of two intensively stained synergids in embryo sacs suggests that the postzygotic phenomenon LSI did not occur. Various sizes (the sizes of the unfertilized ovules differed significantly) of selfed unfertilized ovaries were observed during seed development. Thus, seed abortion occurred at various stages, but the maximal area of unfertilized ovules was much smaller than in normal ovules, which is indicative of a pseudomorphic incidence in unfertilized ovules. In addition, the level of sterility increased 4 weeks AP, which indirectly indicates that self-sterility was not associated with subsequent embryonic development, which started after approximately 3 months of dormancy in *C. oleifera* [9]. And the percentage of abortive ovules 35 days after SP was similar to the final seed abortion rate (the selfed seed set was 10.12%). Additionally, the percentage of abortive ovules remained stable during subsequent development, demonstrating that self-sterility did not occur continuously throughout the life cycle. These results support an LSI-based mechanism. LSI after ovule penetration has been reported in numerous plants [44], [50]. LSI has been documented in *C. sinensis (L.) O. Kuntze*, with selfed tubes penetrating the ovule, but with fewer seed sets after SP compared to CP [51].

Although double fertilization was observed in both SP and CP ovules, with no structural or histological differences, the frequency after SP was much lower than that after CP, which could be attributed to reduced ovule penetration in selfed ovules. According to Rodríguez-Riaño, prezygotic SI is represented by low rates of ovule penetration and double fertilization in selfed ovules compared with crossed ovules [52]. In the present study, the significantly reduced rate of ovule penetration and double fertilization after SP than CP in *C. oleifera* ‘XianglinXLC15′ is suggestive of a prezygotic SI-based mechanism. Similar observations were reported in *Cytisus. multiflorus*, *Ipomopsis aggregate*, and *Tectona grandis* [16], [17], [39]. After double fertilization, the structural characteristics (excluding the area of the embryo sac 130 DAP) were similar between the selfed and crossed ovules. However, consistent suppression of self-unfertilized ovule enlargement AP suggests that self-sterility occurred at the prezygotic stage. This was also observed in *C. multiflorus* and *Cytisus. striatus* [17]. Overall, ovule development is significantly most influenced by the factor of DAP, but not the pollen type.

Sage et al. reported a reduction in fertile ovules in the absence of a stimulus in selfed ovules, which resulted in prezygotic SI [53]. However, stimulation produced by carpel tissue development after CP has been documented in many species [27], [54]–[57]. According to Sage et al., stimulation after CP that resulted in the development of crossed ovules (but not selfed ovules) was detected, indicating that prezygotic abortion in *Narcissus triandrus* was related to post-pollination signaling [53]. A similar phenomenon has been documented in *I. aggregate* [58]. The long-distance signaling that results from self-recognition and self-rejection is lower than in the stigma-ovule in selfed ovaries of *I. aggregate*, and changes in stimulatory function are thought to be caused by this long-distance signaling [16], [59]. Previous studies of long-distance signaling affecting ovule development have been conducted in other plants [54], [60]–[63]. However, in our study, prezygotic recognition and the absence of or change in the stimulus in selfed ovules of *C. oleifera* was not observed. Physiological and histochemical studies of SI and failed ovule penetration in *C. oleifera* based on long-distance signaling in stigma-ovules are currently underway.

### Fruit/seed sets are indicative of reproduction

Our data show that SP significantly reduced fruit/seed sets compared to CP in *C. oleifera*. This was also reported in *I. aggregate* and *Ziziphus* (Chinese jujubes) [58], [64]. Sage et al. hypothesized that this reduced seed set resulted from physiological asynchrony in ovules. Additionally, many hermaphroditic plants were reported to contain a reduced fruit/seed set [26], [39], [65], [66]. In our study, the selfed pollen grains germination and pollen tubes reaching the base of style were observed, similar to CP. The arrival time with them was delayed in SP than CP. After that, lower percentage of ovule penetration after SP than CP was observed and the aborted ovules after SP were greater than those after CP. All these results provided a chain of evidence that the low fruit/seed set after SP treatment was probably due to the delayed time. And this probably occurred between pollen tubes penetrating into the ovaries and the ovule viability. A similar situation was reported in *C. sinensis (L.) O. Kuntze*, another species in Theaceae [51]. However, the fruit sets after OP and SP showed no significant differences. This may be due to self-pollen mixing with cross-pollen in OP, according to other cultivated species [16], [23], [51], [58], [67]. Thus, other cultivars of *C. oleifera* could be planted with *C. oleifera* ‘XianglinXLC15′ to generate an increased fruit/seed set.

Although the fruit/seed set after SP was lower than after CP, the size of the selfed seeds tended to be larger than of the crossed seeds. This was also reported in *C. striatus*, possibly due to greater availability of spatial resources for selfed seeds [17]. It was reported that both pollen resource availability limited fruit set and the number of seeds produced, even the size of seeds in plant [68], [69]. In this study, One or several normal seeds are present in each selfed fruit, compared with the abundant developing seeds found in crossed fruit. The lower level of competition for nutrition and space makes larger seeds possible in selfed fruit of *C. oleifera*. Pollinators are an important factor for seed setting in entomophilous flowers. According to the pollination biology of *C. oleifera*, bees are effective pollinators, but they are seldom active during the flowering phase of *C. oleifera* since the temperature in late autumn is low in southern China [2]. Hence, pollen restriction in *C. oleifera* may have resulted in the low seed set. This has also been reported for *Camellia azalea* [70] and *Cyrtanthus breviflorus* [71].

## Conclusions

In this research, we observed the self-sterility and low fruit/seed set after SP in *Camellia oleifera*, and explained the phenomenon in anatomy and statistics. The results indicated the existence of LSI, which supported by selfed pollen grains could germinated on the stigmas, selfed pollen tube growth in style and ovary, then pollen tubes penetrated into ovules, ovule development and fruit/seed set following SP. Further, significantly lower percentages of ovule penetration and double fertilization following SP than CP, in addition, selfed aborted ovules were prior to zygotes division, preliminary confirmed part of prezygotic LSI existing in *Camellia oleifera*.

## Acknowledgments

The authors thank Professor Zhaokun Wan for the analysis of reproductive biology, Luhong Wang for assistance with field work, and Zhiqiang Han, Hui Xiang, Jing Tang for experimental assistance during the course of work.

## Supporting Information

Figure S1 **Thickness of integument under three fertilization treatments at various days after pollination**.
(TIF)

Figure S2 **Interaction between DAP and FT on IIT**.
(TIF)

Figure S3 **Interaction between DAP and FT on OIT**.
(TIF)
