# Supplementary figures and images for: Self-Sterility in Camellia oleifera May Be Due to the Prezygotic Late-Acting Self-Incompatibility
Source: PLoS One. 2014 Jun 13;9(6):e99639. doi: 10.1371/journal.pone.0099639 (PMC4057179; doi:10.1371/journal.pone.0099639)

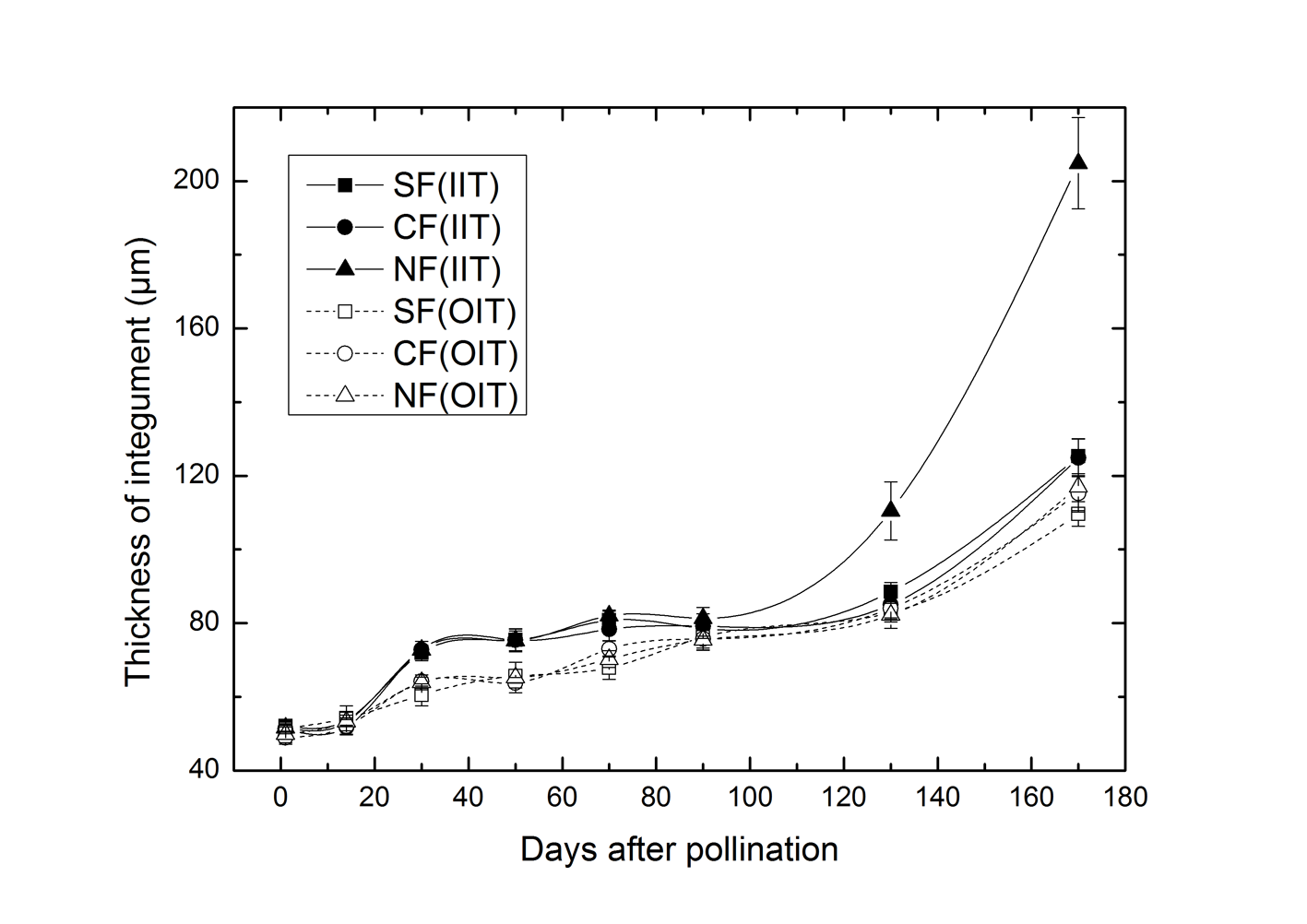

Supplement: Figure S1 — Thickness of integument under three fertilization treatments at various days after pollination. (TIF) [file pone.0099639.s001.tif]

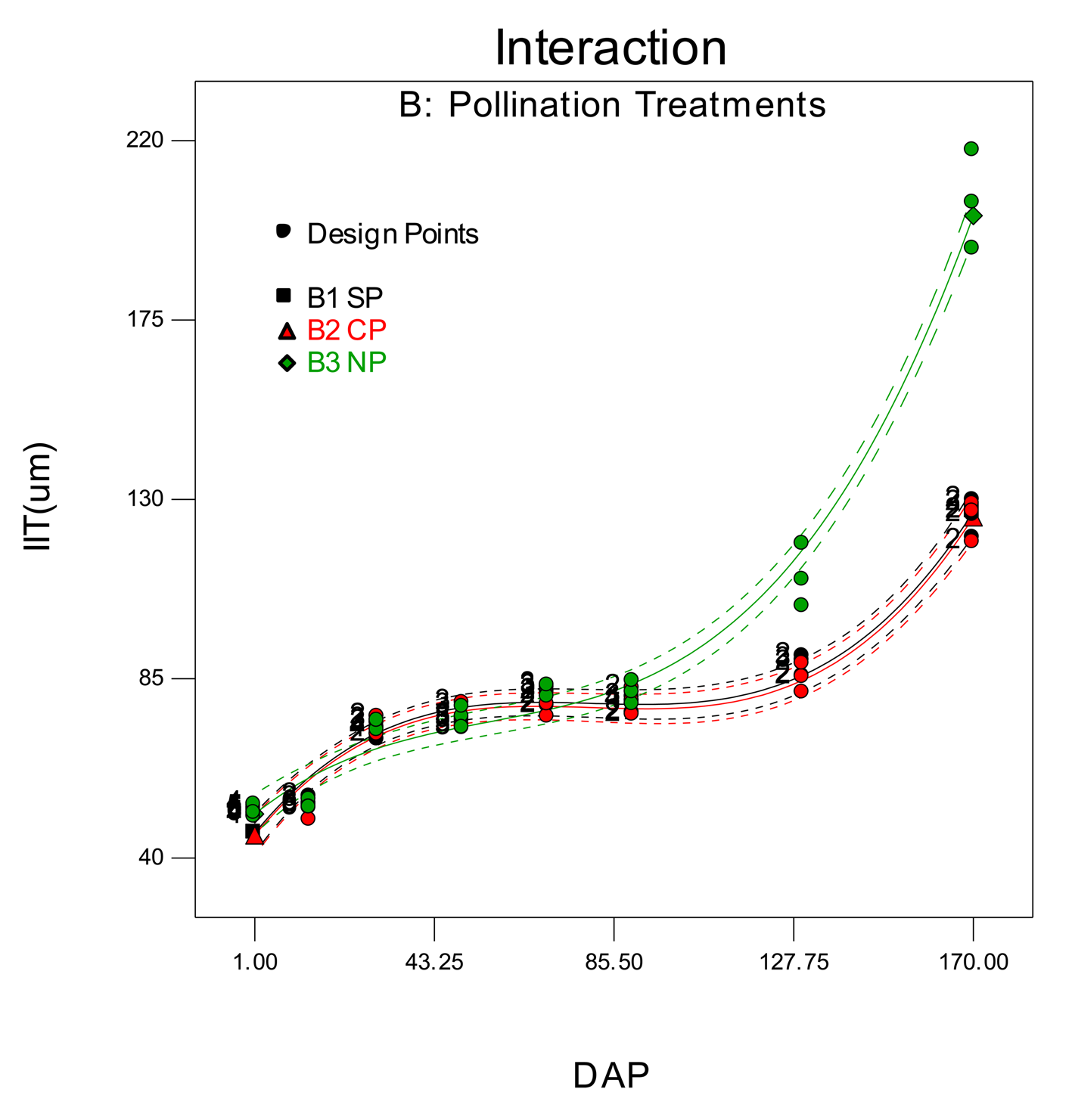

Supplement: Figure S2 — Interaction between DAP and FT on IIT. (TIF) [file pone.0099639.s002.tif]

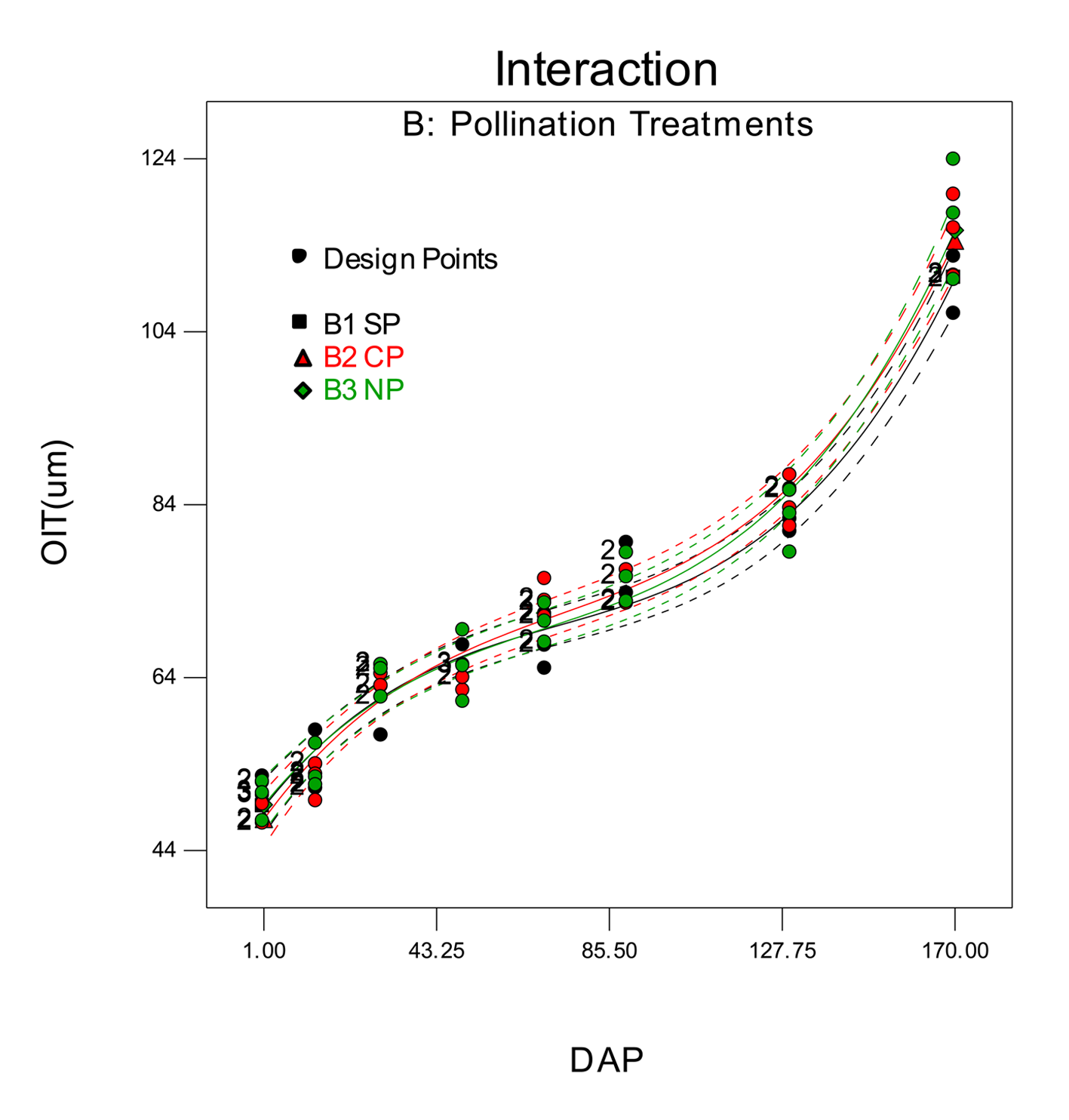

Supplement: Figure S3 — Interaction between DAP and FT on OIT. (TIF) [file pone.0099639.s003.tif]
